# Independent *vs.* Trade Journalism

**Published:** 1889-09

**Authors:** 


### INDEPENDENT *vs.* TRADE JOURNALISM.

In our January number we discussed the claims of the Journal to the support of the profession, and among other things said that ours represented "a journalism that knew no interest except that of the profession, because it was owned and controlled by members of that body." We did not deem it necessary at that time to draw any comparison with other journals, but circumstances over which we have had no control have within the past few months forced us to show wherein some journals, at least, cannot, by reason of their business relations, fairly represent the interests of the profession at large.

When the Dental Protective Association was first organized, Dr. Crouse came to us and allowed us the *privilege*—we use the word advisedly—of bringing the movement before the profession. This we did fully and freely, writing editorials upon the subject, and calling attention to it in each succeeding issue. The matter was also presented to the *Cosmos* with the request that the printed circular should be published with some editorial comment, and at the same time suggesting that a journal which had so long received the support of the profession should feel called upon to present a matter fraught with so much interest to the general body. The question was taken under advisement, and after a time a short extract of the circular appeared without editorial comment, either for or against.

The experience of S. S. White in his fight for the profession with the old Vulcanite Company is no doubt having its influence in shaping the course of the publishers of the *Cosmos* in regard to the present controversy between the Dental Protective Association and the International Tooth Crown Company, which is the immediate successor to the Goodyear Dental Vulcanite Company. We are not at all surprised that the *Cosmos* steers clear of the fight. The position of the house now is very different from what it was at the time of the contest with the rubber company. S. S. White was an individual, and the present management is a corporation. It is well known that corporations have less liberty of action and are generally much more conservative than individuals. Questions of the present character are more apt to be weighed from a dollar-and-cent view. This is so well known that it is a common remark that "corporations have no soul." The company publishers of the *Cosmos* are good business-men and know how to look after their large interests.

They are not engaged in journalism from philanthropic motives,—simply from the love of it; nor do they lose sight of the fact that journalism is only a minor part of their business. With them journalism consists in steering skilfully their craft over the shoals, avoiding as many rocks as possible, for rocks represent customers, and to hit them too hard or too frequently might seriously interfere with the footings up of the ledger at the end of the year. Their main idea is to make as few enemies and to sell as many dollars' worth of goods as possible. An aggressive journalism would be inimical to such a policy and need not be looked for.

In proof of the above statements we cite the course of the *Cosmos* for many years past, and ask when or where has it editorially taken an active part in any of the discussions which have agitated the profession. What the profession needs is an *organ* that shall without fear or favor discuss all questions of a public nature, and form a medium through which the profession may find a voice. The INTERNATIONAL DENTAL JOURNAL offers such a medium, and calls upon the profession to rise and support it heartily. The course of the *Independent Practitioner* has been sufficient evidence in the past that the policy of the JOURNAL has been to discuss all matters of public policy entirely without bias. The past year under our management has been no exception, and we feel safe in saying that we fully understand wherein our duty lies, and shall under all circumstances be found trying to fulfil it to the utmost of our ability.

We are to-day in better shape to lead in dental journalism than ever before. We are fully equipped for the publication of a strictly first-class journal. We have our pick of the productions of the best men in the profession. We are unhampered by business entanglements, which, as we have shown, prevent a journal from expressing an unbiased opinion on subjects of interest to the profession; and lastly, we have the hearty support of the dealers and manufacturers, nearly all of whose advertisements are to be found in our advertising pages. We present to you each month not the goods of one house, but the specialties of all the leading houses of the country. In this latter respect we claim precedence over any other journal published in this country. For the same price charged by other journals we not only present to you the choicest literature of the day, but also bring to you each month all that is new and important in office, laboratory, and shop.

While we accept the advertisements of all reputable houses, yet, by so doing, we do not in any manner sacrifice our independence of expression, but hold ourselves free to criticise any article or house when in our opinion the good of the profession demands it.

